# Educational Case: Phyllodes tumors

**DOI:** 10.1016/j.acpath.2026.100277

**Published:** 2026-07-13

**Authors:** Nicholas Boivin, Ciaran Mannion, Jennifer Zepf

**Affiliations:** a Hackensack Meridian School of Medicine, Nutley, NJ, USA; b Hackensack Meridian University Hospital, Hackensack, NJ, USA

**Keywords:** Pathology competencies, Organ system pathology, Breast, Breast neoplasms, Fibroadenoma, Morphology, Phyllodes tumor, Staging

## Primary objective

The following fictional case is intended as a learning tool within the Pathology Competencies for Medical Education (PCME), a set of national standards for teaching pathology. These are divided into three basic competencies: Disease Mechanisms and Processes, Organ System Pathology, and Diagnostic Medicine and Therapeutic Pathology. For additional information, and a full list of learning objectives for all three competencies, see https://doi.org/10.1016/j.acpath.2023.100086.^1^

Objective BR2.1: Fibroadenoma and Phyllodes Tumors. Compare and contrast fibroadenoma and phyllodes tumors in terms of clinical features, morphologic findings, and prognosis.

Competency 2: Organ System Pathology, Topic: Breast (BR); Learning Goal 2: Breast Neoplasms.

## Secondary objectives

Objective N3.1: Morphologic Features of Neoplasia. Describe the essential morphologic features of neoplasms and indicate how these can be used to diagnose, classify, and predict biological behavior of cancers.

Competency 1: Disease Mechanisms and Processes, Topic: Neoplasia (N); Learning Goal 3: Characteristics of Neoplasia.

Objective N3.5: Grading and Staging of Neoplasia. Compare and contrast the basic grading and staging of neoplastic diseases and describe the tumor, (lymph) nodes, and metastasis classification for common tumors such as breast and colon carcinoma.

Competency 1: Disease Mechanisms and Processes; Topic: Neoplasia (N); Learning Goal 3: Characteristics of Neoplasia.

## Patient presentation

A 41-year-old woman presents to the clinic after feeling a small lump in her right breast. She has not observed skin changes or nipple discharge. She otherwise feels healthy and has no recent history of trauma. Her past medical history includes hypercholesterolemia and obesity. She takes no medications. Physical examination reveals a firm, nontender lump in the upper-outer quadrant of the right breast approximately 3 cm in size. There is no nipple discharge, skin changes, or lymphadenopathy appreciated on examination and she is afebrile.

## Diagnostic findings, Part 1

**Fig. 1:**
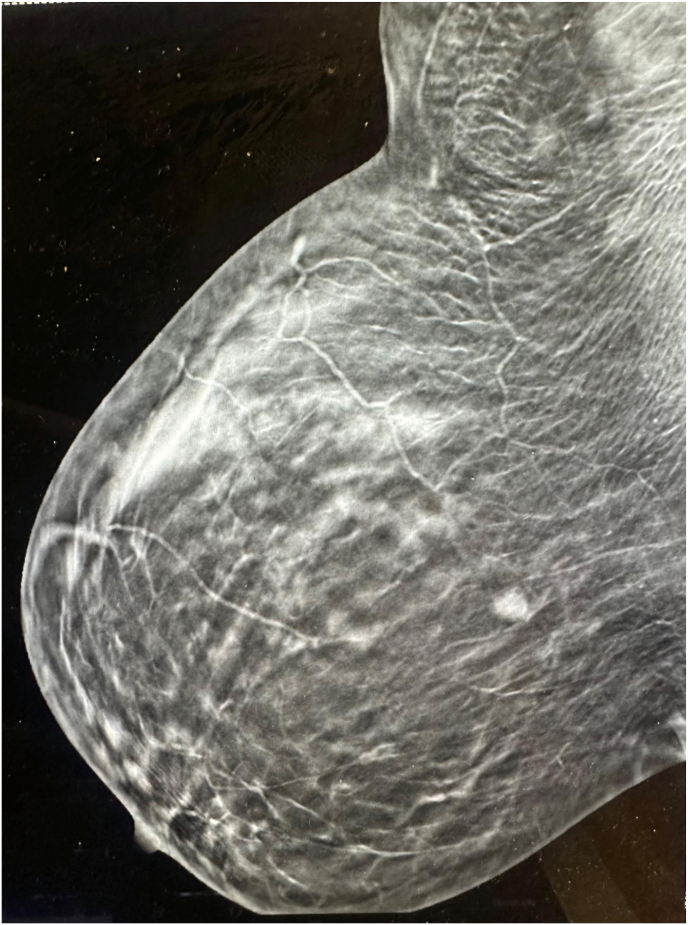
Mammogram of the right breast nodule showing calcifications in the right upper-outer portion of the breast.

Fig. 1 shows the patient’s right mammogram from six months earlier. Findings include bilateral calcifications of indeterminate significance, with the breast showing a mixture of fatty and dense fibrous tissues. Based on these images, the patient was assigned a breast imaging-reporting and data system (BI-RADS) category of 0.

## Questions/discussion points, Part 1

### What should be considered in the differential diagnosis?

The differential for a new breast mass will include a cyst, fat necrosis, a fibroepithelial lesion, an abscess, and a malignant neoplasm. Both fibroepithelial lesions, including both a phyllodes tumor or a fibroadenoma, and cysts can present as a unilateral nontender breast mass. Although the patient lacks alarm symptoms, such as lymphadenopathy, skin changes, and nipple discharge, malignancy is still possible and must be investigated. Some less likely differentials include a breast abscess (may present with a unilateral breast mass but the mass is often tender and patients experience fever and malaise) and fat necrosis (usually has associated skin ecchymosis and a history of trauma to the chest).

### How is imaging used to evaluate a breast mass?

Radiographic evaluation of a mammogram or breast ultrasound primarily assesses the breast tissue for density, masses, calcifications, asymmetries, associated features, and location of any lesions.^2^ If any masses are found, the radiologist looks at other factors (such as shape and density) to determine the risk of malignancy. A mass that is round with circumscribed borders is likely to be benign, whereas a dense mass with an irregular contour is more likely to be malignant. Calcifications, when present, can also be analyzed as to the risk of malignancy. Benign calcifications are often described as being popcorn or ‘rod-like’, whereas those exhibiting linear branching are more likely to be malignant.

Once the radiologist has completed the evaluation, the findings are classified to a BI-RADS category, ranging from 0 to 6. The score is determined based on the risk of malignancy with a score of 1 corresponding to a risk of malignancy near 0% and a score of 5 corresponding to a risk of malignancy over 95%. A score of 0 means that the radiologist was not able to appropriately determine the malignancy risk and a score of 6 means there has been a previously confirmed malignancy based on histologic examination. Patient management varies based on the determined BI-RADS score. The different BI-RADS categories, their associated malignancy risk, and their management are described in Table 1.

**Table 1:** BI-RADS categories and their corresponding findings, malignancy risk, and management.^2^

BI-RADS category	Category	Malignancy Risk	Management
0	Incomplete	Unknown	Follow-up imaging
1	Negative	∼0%	Routine screening
2	Benign	∼0%	Routine screening
3	Likely benign	<5%	Short-interval follow-up
4	Suspicious	5–95%	Biopsy
5	Likely malignancy	>95%	Biopsy
6	Histologically proven malignancy	Previously histologically confirmed malignancy	Treatment planning

BI-RADS: breast imaging-reporting and data system.

### What should be the next diagnostic study performed and why?

Based on the patient’s BI-RADS category 0 classification, her last mammogram was indeterminate. Given the presence of areas of dense breast tissue in the prior mammogram, ultrasonographic evaluation would be the preferred imaging modality; unlike mammography, ultrasonography has been established to have increased sensitivity and specificity for detecting breast cancer in patients with dense breast tissue.^3^^,^^4^

## Diagnostic findings, Part 2

**Fig. 2:**
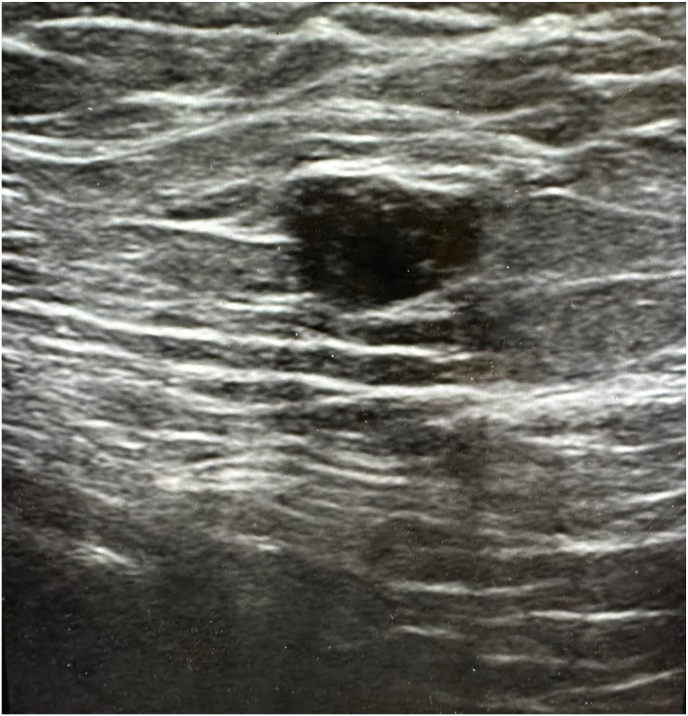
Ultrasound of the right breast.

The patient undergoes ultrasound, with findings from the right breast shown in Fig. 2.

## Questions/discussion points, Part 2

### How should the ultrasound in Fig. 2 be interpreted?

There is a nodule that appears to be hypoechoic, oval shaped, and with well-circumscribed borders.

### Why is the description of the nodule important?

The nodule is hypoechoic, ovoid, and has well-circumscribed borders. Although the shape and borders of this nodule suggests a benign process, hypoechoic nodules have a higher risk of malignancy than hyperechoic nodules, with the former typically of solid consistency, whereas hyperechoic masses are usually fluid-filled lesions, such as cysts.^5^

### What BI-RADS category would you expect to be given in this case?

The nodule has a mix of benign (ovoid shape and well-circumscribed borders) and concerning findings (hypoechoic). Because of these mixed findings, the nodule is suspicious with the risk of malignancy falling between 2% and 95%. This would place the patient into BI-RADS category 4.

### What is the next best step in management?

According to the American College of Radiology, when a BI-RADS of 4 or 5 is assigned, the next step is to biopsy the lesion.^6^

## Diagnostic findings, Part 3

**Fig. 3:**
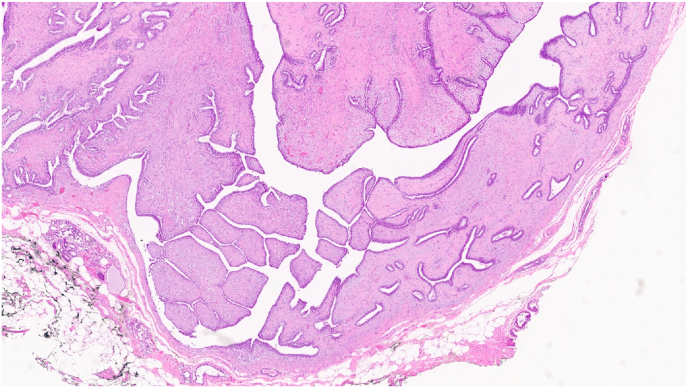
Breast excision, H&E, 2x. H&E: hematoxylin and eosin.

**Fig. 4:**
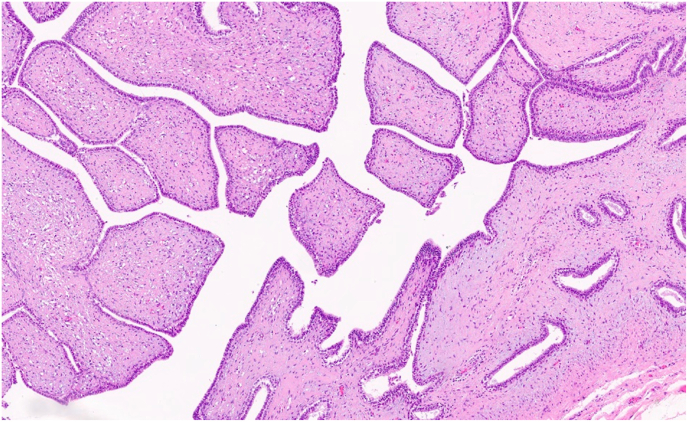
Breast excision, H&E, 4x. H&E: hematoxylin and eosin.

A core needle biopsy was performed and interpreted to be a fibroepithelial lesion. Fibroepithelial lesions are biphasic, meaning there is proliferation of two cell types, in this case, both the stroma and the epithelium. There are two tumor types that fall under the umbrella of fibroepithelial tumors, fibroadenomas and phyllodes tumors. Fibroadenomas are almost always benign. Phyllodes tumors are graded as benign, borderline, or malignant. It is exceptionally challenging to differentiate fibroadenomas and phyllodes tumors from a biopsy. Because one cannot safely rule out a malignant phyllodes tumor based on a core needle biopsy, findings of a fibroepithelial lesion on biopsy necessitate an excision for further workup. This patient undergoes an excision revealing the histologic findings in Fig. 3, Fig. 4.

## Questions/discussion points, Part 3

### What are the histologic findings of the breast excision demonstrated in Fig. 3, Fig. 4?

There is an intracanalicular “slit-like” growth pattern, creating a leafy architecture with central light pink stroma and a surrounding peripheral layer of purple epithelial cells. Stromal cellularity is mildly increased, without prominent mitotic figures.^7^ Tumor borders appear well defined. There is no stromal overgrowth (defined as an expansion of the stroma to such an extent that there is an absence of epithelial elements in a low-power microscopic field) and minimal-to-mild stromal cytologic atypia. Stromal fragmentation, an architectural arrangement in which there appears to be free-floating chunks of stromal tissue completely rimmed by a peripheral epithelial cell layer, is a sign of extreme intracanalicular growth pattern and may also be seen in phyllodes tumors.^8^

### Based on these histologic findings, what is the most likely diagnosis and stage?

In this case, the patient most likely has a benign phyllodes tumor. Benign phyllodes tumors represent approximately 60%–75% of all phyllodes tumors.^9^ Unlike invasive breast carcinomas (also called malignant epithelial tumors), phyllodes tumors of the breast lack a specific patient staging system. Only malignant phyllodes tumors are staged in accordance with the American Joint Committee on Cancer staging rules for sarcomas of the extremity and trunk (malignant mesenchymal tumors).^10^^,^^11^

### What are the genetic risk factors and epidemiology for phyllodes tumors?

Phyllodes tumors tend to present in middle-aged women, most often between the ages of 45–49 years.^12^ They are rare, with a reported incidence of 2.53 per 100,000 women receiving breast screening.^13^ There is an association with Li–Fraumeni syndrome and mutations in *MED12* and *TERT*.^7^^,^^14^^,^^15^ *MED12* encodes a member of the multiprotein mediator complex with mutations occurring in various (often hormone sensitive) tumor types. Of note, *MED12* mutations are most often seen in benign and borderline phyllodes tumors and less so in malignant phyllodes tumors. *TERT* encodes a subunit of telomerase with its transcription being activated in numerous malignant tumors.

### If the tumor was malignant, what histologic findings would be present?

Only, approximately, 9% of phyllodes tumors are malignant. However, the likelihood of malignancy is significantly increased in patients presenting with large tumors. Phyllodes tumors over 10 cm at the time of diagnosis have a 42.5% likelihood of being malignant.^16^^,^^17^ Malignant phyllodes tumors display pronounced stromal hypercellularity and conspicuous stromal cytologic atypia, with nuclear pleomorphism. The high proliferation rate of the neoplastic stromal cells, with a mitotic count often in excess of 10 mitotic figures per 10 high-power fields (HPFs), is frequently accompanied by marked lesional stromal expansion, resulting in stromal overgrowth, with proportionately reduced epithelial elements. The tumor borders are often poorly defined and infiltrative in character.

**Fig. 5:**
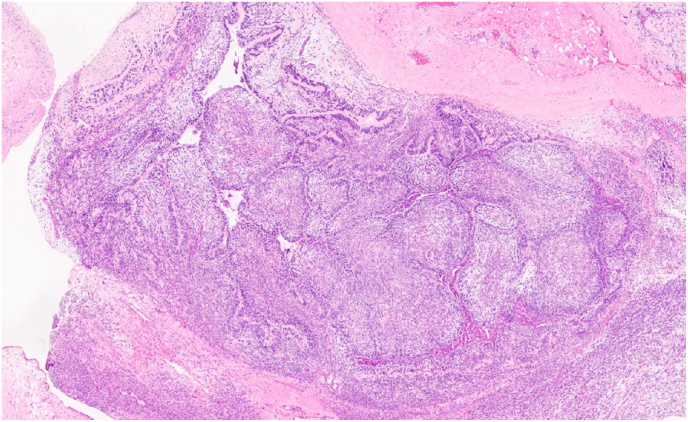
Malignant phyllodes tumor with hypercellular stroma. H&E, 2x. H&E: hematoxylin and eosin.

**Fig. 6:**
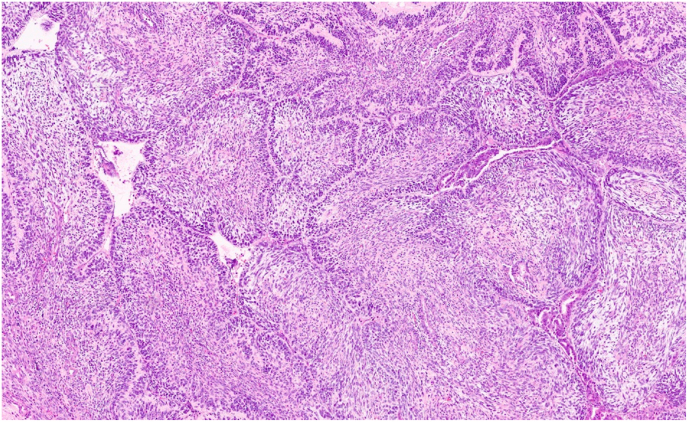
Malignant phyllodes tumor with hypercellular stroma. H&E, 4x. H&E: hematoxylin and eosin.

**Fig. 7:**
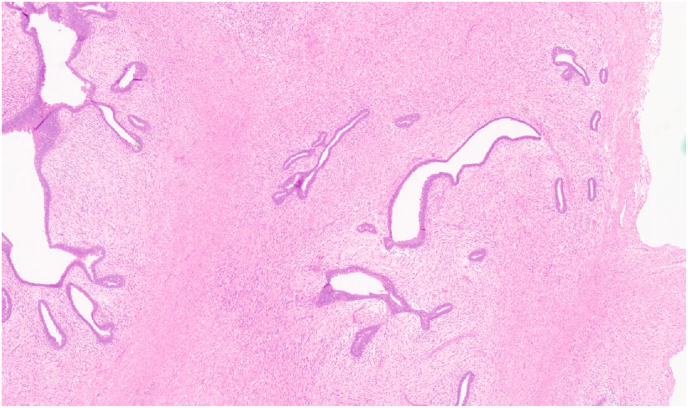
Malignant phyllodes tumor with significant stromal expansion. H&E, 2x. H&E: hematoxylin and eosin.

**Fig. 8:**
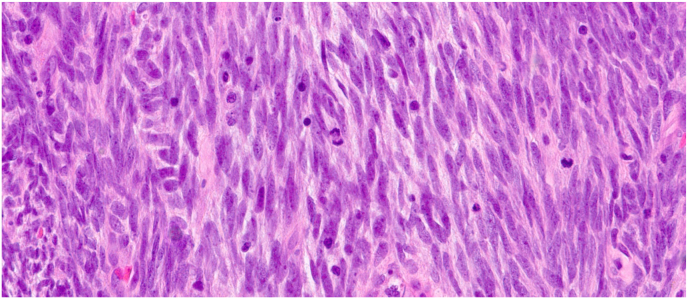
Malignant phyllodes tumor with numerous mitotic figures. H&E, 40x. H&E: hematoxylin and eosin.

Fig. 5, Fig. 6 show low-power histologic images of a malignant phyllodes tumor demonstrating hypercellular stroma. Fig. 7 is also a low-power image of a malignant phyllodes tumor with significant stromal expansion. Fig. 8 shows a high-power image of a malignant phyllodes tumor demonstrating several mitotic figures (including one that is particularly conspicuous, just left of center) within extremely cellular stroma with a high nuclear-to-cytoplasmic ratio.

### If the tumor was a borderline phyllodes tumor, what histologic findings would be present?

Borderline tumors have features that are intermediate between malignant and benign phyllodes tumors. They constitute an estimated 12–26% of all phyllodes tumors.^9^ The tumor borders should, for the most part, be relatively well circumscribed, with relative focal invasion into the surrounding peritumoral tissue. Nuclear pleomorphism is moderate. Compared to benign phyllodes tumors, borderline tumors have more stromal atypia and greater stromal cellularity that may result in some focal stromal overgrowth.^18^ Mitotic figures are between 5 and 9 per 10 HPF.

**Fig. 9:**
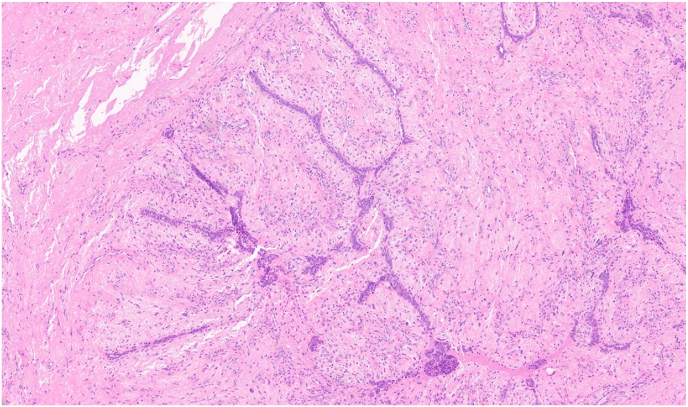
Borderline phyllodes tumor showing hypercellular stroma, although not as pronounced as seen in Fig. 5, Fig. 6 for a malignant phyllodes tumor. H&E, 4x. H&E: hematoxylin and eosin.

**Fig. 10:**
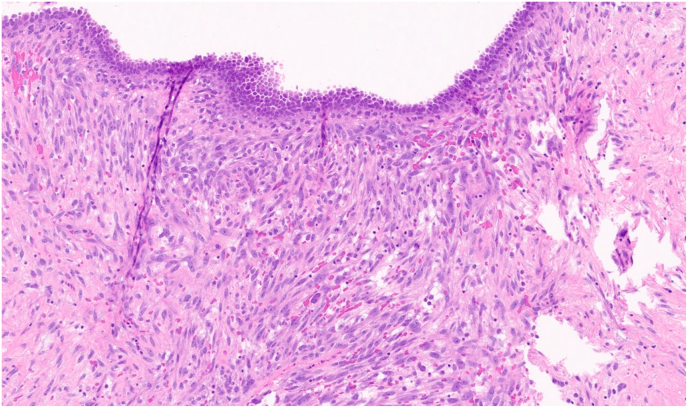
Borderline phyllodes tumor with hypercellular stroma. H&E, 10x. H&E: hematoxylin and eosin.

**Fig. 11:**
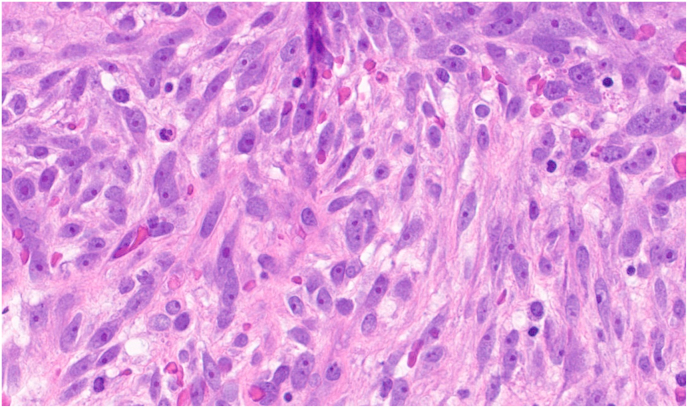
Borderline phyllodes tumor with enlarged, cytologically atypical stromal cells. However, compared to the malignant phyllodes tumor in Fig. 9, there are less mitotic figures present. H&E, 40x. H&E: hematoxylin and eosin.

Fig. 9, Fig. 10, and Fig. 11 show histologic images of borderline phyllodes tumors. In Fig. 10, the stromal hypercellularity is readily apparent, while in Fig. 11, enlarged and cytologically atypical stromal cells are evident.

### Are there any immunostains that can help when grading a phyllodes tumor?

Immunohistochemical stains have a minimal role to play in the evaluation of phyllodes tumors. The percentage of neoplastic stromal cells demonstrating nuclear staining with Ki67, a proliferation marker, typically mirrors mitotic rates observed on routine hematoxylin and eosin (H&E) stained slides. The intensity of Ki67 staining increases with tumor grade, with benign tumors exhibiting weak staining and malignant tumors strongly staining.^19^ As a rule, for phyllodes tumors, the utility of Ki67 staining for grading is not absolute. In the event of a contradiction, the histologic findings on routine H&E staining should always take precedence over the Ki67 stain results.

### What is the prognosis for phyllodes tumors?

As a whole, phyllodes tumors have a 10-year survival rate of 87%.^20^ The 5-year no evidence of disease survival rate is estimated to be 95.7% in patients with benign phyllodes tumors, 73.7% for borderline phyllodes tumors, and 66.1% for malignant phyllodes tumors.^21^ Recurrence is common for any grade of phyllodes tumor. For benign phyllodes tumors, the estimate is 10–17%; for borderline tumors , the estimate is 14–25%; and for malignant tumors, the rate of recurrence is 23–30%.

Benign phyllodes tumors have no risk of metastasis. Malignant transformation occurring in a preexisting benign phyllodes tumor is rare, with only a handful of case reports.^22^ Borderline phyllodes tumors can metastasize, although it is exceptionally uncommon. Malignant phyllodes tumors metastasize in 3–12% of cases.^7^ As phyllodes tumors spread hematogenously, in contrast to invasive carcinomas, metastatic involvement of axillary lymph nodes from an ipsilateral malignant phyllodes tumor is rare.^17^ Given the mesenchymal nature of these tumors, it is not surprising that the most common location for metastasis is the lungs, followed by bone. When they do metastasize, patients often die within 3 years due to the poor response of malignant phyllodes tumors to chemotherapy.^9^ The larger the phyllodes tumor, the more likely it is to recur and deaths from phyllodes tumors are exceptional in those that are less than 5 cm at the time of diagnosis.^23^

### How are phyllodes tumors treated?

Phyllodes tumors are traditionally treated with wide local excision.^24^ Margins should be at least 1 cm tumor free for borderline and malignant phyllodes tumors.^25^ Benign phyllodes tumors are excised to remove the mass without consideration of margins.^26^ Mastectomy may be required if the tumor is large.^24^ Axillary lymph node dissection is usually not done since there is rarely any involvement. Endocrine and adjuvant chemotherapy are generally administered. Adjuvant radiation may sometimes be deployed if negative margins are unable to be achieved on excision. When phyllodes tumors recur, they nearly always do so within the first 24 months after initial surgery. After excision, patients should be evaluated for recurrence every 6 months for the first 2 years with ultrasound, then annually for an additional 3 years.^26^ These evaluations are in addition to annual mammograms as per current guidelines. For benign phyllodes tumors, it is reasonable to shorten the follow-up period to 3 years total, with imaging and time between visits at the clinician’s discretion. If there is high concern for recurrence, follow-ups can be more frequent and include chest radiographs and/or chest computed tomography to assess for possible hematogenous metastasis of tumor to the lungs.

### What other fibroepithelial tumor should be considered and what would be the differences in epidemiology, etiology, prognosis, and management??

The more common fibroepithelial tumor is a fibroadenoma. This is a benign breast tumor, usually presenting in patients between the ages of 20 and 35 years, most often as a unilateral mass.^7^ Prevalence is approximately 27.6% in women aged 18–40 years old.^27^ Fibroadenomas are estrogen and progesterone receptive. This is why they are most common in premenopausal women and women who are pregnant. Therefore, patients with fibroadenomas tend to be younger than those with phyllodes tumors.^7^^,^^12^ It should also be noted that both fibroadenomas and phyllodes tumors are rare in males, primarily described in case reports. Rarely, either lesion can arise from persistent breast tissue at any point along an incompletely resolved, embryologic milk line that extends from the axilla to the vulva.

Prognosis for fibroadenomas is favorable, with only about 1/1000 cases having malignant changes. Management is typically conservative, with excision rarely necessary.^28^ If they are removed, recurrence is rare. Similar to phyllodes tumors, fibroadenomas are associated with *MED12* mutations but they are not as strongly associated with *TERT* mutations.^14^^,^^15^

### How do fibroadenomas and phyllodes tumors differ based on their gross appearances?

Grossly, fibroadenomas are small, white, ovoid in shape, with a firm or rubbery feel.^7^ They rarely grow larger than 4 cm. The cut surface is solid, grayish white, with slit-like spaces. There should not be any necrosis. Similarly, a phyllodes tumor is usually round in shape, a whitish color, and feels firm. Phyllodes tumors are usually larger, often with a clinical history of rapid growth and, due to the higher proliferation rate, can occasionally display necrosis or hemorrhaging. The cut surface is solid, grayish white, and has cleft-like spaces that resemble the leaf-like pattern seen microscopically. Because fibroadenomas grow slowly or not at all, monitoring the size of a lesion over time can help differentiate fibroadenomas and phyllodes tumors. Although phyllodes tumors and fibroadenomas generally appear in different age groups and usually differ in size grossly, there are exceptions. Therefore, these factors alone are unreliable for diagnosis.

### Fig. 12, Fig. 13 show low-power images of a fibroadenoma. Based on your interpretation of these figures, how do fibroadenomas and phyllodes tumors differ histologically?

**Fig. 12:**
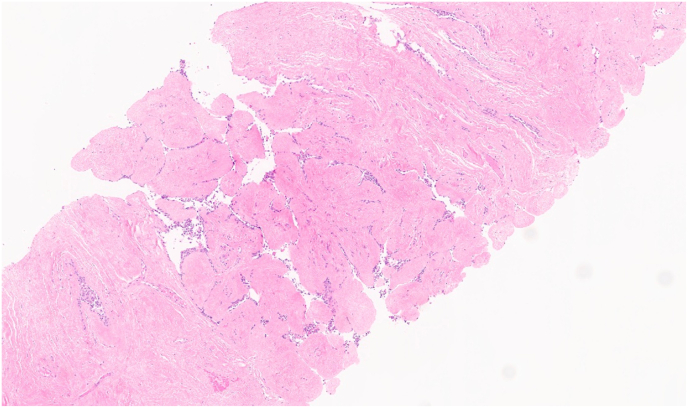
Fibroadenoma with stroma lacking cellularity and no visible mitotic figures. H&E, 4x. H&E: hematoxylin and eosin.

**Fig. 13:**
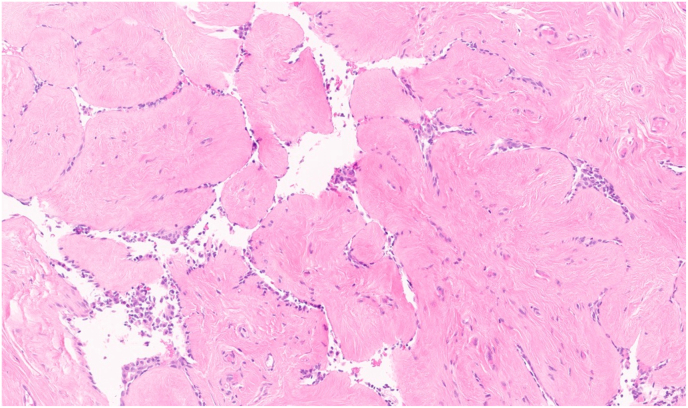
Fibroadenoma with stroma lacking cellularity and no visible mitotic figures. H&E, 10x. H&E: hematoxylin and eosin.

Histologically, fibroadenomas are well-circumscribed lesions with a uniform growth pattern of stromal and glandular elements.^7^ Glands can grow in an intracanalicular pattern where they appear similar to moose antlers with linear branching structures or in a pericanalicular pattern where glands retain open lumens but are separated by expanded stroma. They have few, if any, mitotic figures. The stroma has low cellularity without atypia (however, it should be noted that there is a variant known as cellular fibroadenoma in which the stroma does have increased cellularity). These features are present in Fig. 12, Fig. 13. There may also be multinucleated giant cells present in the stroma.

However, fibroadenomas can appear similar to phyllodes tumors. This is especially true when differentiating a benign phyllodes tumor with few mitoses and atypia from a cellular fibroadenoma that can have increased cellular stroma similar to a phyllodes tumor. Additionally, phyllodes tumors can be heterogeneous in appearance, with large sections of the lesion appearing similar to a fibroadenoma. Therefore, in a core needle biopsy sampling where only part of the lesion is being sampled and microscopically examined, it can be exceptionally challenging to differentiate these fibroepithelial lesions.

Nevertheless, there are several histologic features that can assist in differentiating phyllodes tumors from fibroadenomas. Mitotic activity, specifically three or more mitotic figures per 10 HPF, is far more likely to be encountered in a phyllodes tumor.^8^ Stromal cytologic atypia, stromal overgrowth, stromal fragmentation, and stromal infiltration of adipose tissue at the periphery of the lesion are all features favoring a phyllodes tumor. Although stromal cellularity has low utility in differentiating cellular fibroadenoma from phyllodes tumors, subepithelial stromal hypercellularity (often called “stromal condensation”) suggests a phyllodes tumor. In addition, while the heterogeneity of phyllodes tumors makes it difficult to evaluate them on core needle biopsy, if lesional heterogeneity is evident within the sampling, this observation is more typical of a phyllodes tumor rather than a fibroadenoma. Information summarizing how to differentiate fibroadenomas from each grade of phyllodes tumor is shown below in Table 2.

**Table 2:** Histologic feature comparison of fibroadenoma and the three grades of phyllodes tumor.^7^^,^^8^^,^^18^

	Fibroadenoma	Benign phyllodes	Borderline phyllodes	Malignant phyllodes
Stromal atypia	None	Mild	Moderate	High
Stromal cellularity	Normal; rarely mildly increased (without condensation)	Mildly increased (with condensation)	Moderately increased	Significantly increased
Mitotic count	0–2/10 HPF	<5/10 HPF	5–9/10 HPF	10/10 HPF or greater
Tumor border	Well circumscribed	Well circumscribed	Well circumscribed with occasional focal invasion	Poorly defined with significant infiltration

HPF: high-power field.

## Teaching points

• Phyllodes tumors are fibroepithelial tumors that usually present in middle-aged women, while fibroadenomas are often found in women of reproductive age since they are hormonally responsive.
• Fibroepithelial tumors feature biphasic proliferation of both stroma and epithelium, and include fibroadenomas and phyllodes tumors as subtypes.
• Although fibroadenomas and phyllodes tumors can appear similar histologically, the presence of mitotic figures, stromal overgrowth, stromal infiltration into adipose tissue, stromal fragmentation, subepithelial stromal condensation, stromal nuclear atypia/pleomorphism, and stromal heterogeneity are all features favoring a phyllodes tumor.
• Due to the difficulty of differentiating fibroadenomas and phyllodes tumors on core needle biopsies, a biopsy that contains a fibroepithelial tumor may necessitate an excision for further workup.
• Both fibroadenomas and phyllodes tumors often have mutations in MED12.
• Phyllodes tumors are graded as benign, borderline, or malignant, depending on their histologic features, including mitotic activity, the degree of stromal hypercellularity and stromal cytologic atypia, the presence of stromal overgrowth, and the integrity (smooth versus infiltrative) of the tumor border with the perilesional tissue.
• Breast imaging-reporting and data system (BI-RADS) is a scoring system, ranging from 0 to 6, that is used by radiologists to estimate the likelihood of a breast malignancy.
• A BI-RADS score of 4 or 5 necessitates a biopsy for microscopic tissue examination by a pathologist to assess for malignancy.
• Ultrasound imaging of the breast can be useful in patients with inconclusive findings and/or dense tissue on their mammogram.

## Funding

The article processing fee for this article was funded by an Open Access Award given by the Society of ‘67, which supports the mission of the Association for Academic Pathology to produce the next generation of outstanding investigators and educational scholars in the field of pathology. This award helps to promote the publication of high-quality original scholarship in *Academic Pathology* by authors at an early stage of academic development.

## Declaration of competing interest

The author(s) declared no potential conflicts of interest with respect to the research, authorship, and/or publication of this article.
